# CBAP Functions as a Novel Component in Chemokine-Induced ZAP70-Mediated T-Cell Adhesion and Migration

**DOI:** 10.1371/journal.pone.0061761

**Published:** 2013-04-19

**Authors:** Yun-Jung Chiang, Kun-Chin Ho, Chien-Tsang Sun, Jeng-Jiann Chiu, Fang-Jen Lee, Fang Liao, Hsin-Fang Yang-Yen, Jeffrey Jong-Young Yen

**Affiliations:** 1 Institute of Microbiology and Immunology, National Yang-Ming University, Taipei, Taiwan; 2 Institute of Biomedical Sciences, Academia Sinica, Taipei, Taiwan; 3 Institute of Molecular Biology, Academia Sinica, Taipei, Taiwan; 4 Division of Medical Engineering Research, National Health Research Institutes, Miaoli, Taiwan; 5 Institute of Molecular Medicine, College of Medicine, National Taiwan University, Taipei, Taiwan; 6 Taiwan Mouse Clinic, Academia Sinica, Taipei, Taiwan; Baylor College of Medicine, United States of America

## Abstract

Activated chemokine receptor initiates inside-out signaling to transiently trigger activation of integrins, a process involving multiple components that have not been fully characterized. Here we report that GM-CSF/IL-3/IL-5 receptor common beta-chain-associated protein (CBAP) is required to optimize this inside-out signaling and activation of integrins. First, knockdown of CBAP expression in human Jurkat T cells caused attenuated CXC chemokine ligand-12 (CXCL12)-induced cell migration and integrin α4β1- and αLβ2-mediated cell adhesion *in vitro*, which could be rescued sufficiently upon expression of murine CBAP proteins. Freshly isolated CBAP-deficient primary T cells also exhibited diminution of chemotaxis toward CC chemokine ligand-21 (CCL21) and CXCL12, and these chemokines-induced T-cell adhesions *in vitro*. Adoptive transfer of isolated naive T cells demonstrated that CBAP deficiency significantly reduced lymph node homing ability *in vivo*. Finally, migration of T cell-receptor–activated T cells induced by inflammatory chemokines was also attenuated in CBAP-deficient cells. Further analyses revealed that CBAP constitutively associated with both integrin β1 and ZAP70 and that CBAP is required for chemokine-induced initial binding of the talin-Vav1 complex to integrin β1 and to facilitate subsequent ZAP70-mediated dissociation of the talin-Vav1 complex and Vav1 phosphorylation. Within such an integrin signaling complex, CBAP likely functions as an adaptor and ultimately leads to activation of both integrin α4β1 and Rac1. Taken together, our data suggest that CBAP indeed can function as a novel signaling component within the ZAP70/Vav1/talin complex and plays an important role in regulating chemokine-promoted T-cell trafficking.

## Introduction

Chemokine/chemokine receptor–mediated signals are implicated in regulating trafficking, which is important for maintaining homeostasis in peripheral lymphoid organs and triggering immune responses after infection. Upon entry of T lymphocytes into peripheral lymphoid tissues, especially lymph nodes (LNs) and Peyer’s patches, the process is tightly controlled by interaction between T cells and specialized vessels known as high endothelial venules, which express CCL21, a ligand for the homeostatic CC-chemokine receptor CCR7 [1], [2]. Although L-selectin and integrins are the major adhesion molecules expressed on the surface of T cells, signals from homeostatic chemokine receptors, such as CCR7 [3], [4], are absolutely required for the process of stable adhesion and migration of T lymphocytes towards lymphoid organs via high endothelial venules [5]–[7].

Chemokine receptors, members of the G protein–coupled receptor family, transduce diverse signals to regulate many functions during the recruitment of leukocytes from the blood stream; these processes include cell polarization, migration, and adhesion. Among the signaling molecules involved, ZAP70, a T lymphocyte–specific Syk family kinase, was recently reported to play an important role in T-cell transendothelial migration [8] and directional chemotaxis *in vitro* [9] and to critically regulate integrin-dependent adhesion via control of phosphorylation of the guanine nucleotide exchange factor, Vav1, which is specific for the small Rho family GTPase Rac1; this phosphorylation causes dissociation of the Vav1-talin signal complex [10]. Consistent with these reports, Vav1 has been demonstrated to play an important role in CXCL12-induced cell adhesion via promoting generation of Rac1-GTP [11] and T-cell chemotactic activity *in vitro* [12]. Rac1 is critical for transducing chemokine signals for regulation of T-lymphocyte motility and adhesion [13]. As one downstream target of Vav1, Rac1 is rapidly activated upon stimulation by CXCL12 [14]. Overexpression of the constitutively active mutant RacV12 in a T-cell line enhances cell spreading and adhesion [15], whereas expression of a dominant-negative mutant of Rac1, or Rac1-specific small interfering RNA, reduces CXCL12-induced transendothelial migration [8] and adhesion in T-cell lines and human T lymphocytes [11].

Rac1 and another Rho family small GTPase, Cdc42, both act in a similar pathway at the leading edge of polarized T cells through the WASP (Wiskott-Aldrich syndrome protein) and WAVE (WASP-family verprolin homologous protein) proteins to promote actin nucleation for dramatic cytoskeletal rearrangement, which is required for chemokine-induced lymphocyte polarization and movement [16]. On the other hand, integrins are a large family of transmembrane heterodimers consisting of one α- and one β-subunit, which is required for chemokine-induced firm adhesion of T cells. Resting T cells bind integrin ligands with low affinity, but these ligands can bind with high affinity after stimulation with chemokines, which alters the integrin conformation [17]. For chemokine-induced activation of integrins (i.e., inside-out signaling), binding of talin to the cytoplasmic tail of the integrin β-subunit is a critical step [18], [19] because talin knockdown in T cells results in near-complete loss of integrin-dependent cell adhesion [10]. Talin is a homodimeric protein that can physically link integrin β subunits with the actin cytoskeleton [19]–[21]. Chemokine-stimulated activation of the type I phosphatidylinositol phosphate kinase γ90 increases the generation of phosphatidylinositol 4,5-bisphosphate [11], [22], which results in a conformational change in talin and a subsequent increase in its affinity for the integrin β1 subunit [23], [24]. Chemokine-induced dissociation of Vav1 from the Vav1-talin complex after tyrosine phosphorylation of Vav1 by ZAP70 leads to a further increase in the binding affinity of newly released talin for integrin β1, which results in further α4β1 integrin activation and strengthens cell adhesion [10].

Recently, it was reported that the GM-CSF/IL-3/IL-5 receptor common β chain (βc) directly interacts with integrin β1 and plays an important role during vasculogenesis and tumor angiogenesis [25], [26]. Our earlier study identified a novel transmembrane protein, the GM-CSF/IL-3/IL-5 receptor common beta-chain-associated protein (CBAP), from a human lymphocyte cDNA library using the yeast two-hybrid screen with the cytoplasmic domain of βc as the bait [27]. Upon cytokine deprivation, CBAP could interact directly with the unliganded βc subunit and accelerate apoptosis through a mitochondria-dependent pathway [27]. Therefore, we investigated whether there is a functional link among βc, CBAP, and the integrin β1 complex. Here, we report that CBAP plays an important role in T-cell migration and adhesion and propose a possible mechanism.

## Materials and Methods

### Animals and Genotyping

The gene targeting strategy for CBAP is described in supporting Figure S1A. The *CBAP* knockout (*CBAP* ^−/−^) mice were generated by conventional gene targeting technology. Mouse genotyping was carried out by polymerase chain reaction (PCR) using the following three primers (P1–3, Figure S1A) that generated 785- and 440-bp DNA fragments for the wild-type (WT) and CBAP knockout allele, respectively: P1 (5′-CTGAGTCAGGTGAACCGATTTACC-3′), P2 (5′-CCAAAATTCCACGCATCAGTTCTG-3′), and P3 (5′-TAACCGTGCATCTGCCAGTTTGAG-3′). The primers used for reverse transcription polymerase chain reaction (RT-PCR) analysis of CBAP mRNA were as follows: forward, 5′-CAGCTGCAGGAGCTCACTCAGTTA-3′; reverse, 5′-CCATGCAGAACAGGATTCCAGATC-3′.

*CBAP* ^−/−^ mice were backcrossed for more than 10 generations to the C57BL/6 background. This study was carried out in strict accordance with the recommendations in the Guide for the Care and Use of Laboratory Animals of the National Institutes of Health. The protocol was approved by Institutional Animal Care and Utilization Committee (IACUC) of Academia Sinica (approved protocol number: MMiIBMYJ2010084). Mice were euthanized with carbon dioxide and all efforts were made to minimize suffering.

### Plasmids and Reagents

The integrin β1 expression vector was purchased from Addgene (Cambridge, MA). ZAP70 cDNA was purchased from Addgene and was subsequently subcloned into the pCMV-tag3B vector to acquire the Myc-tag. GFP-tagged deletion constructs of CBAP (M1–M5) were all constructed using the pEGFP-N3 expression vector. The glutathione-S-transferase (GST)-p21-binding domain (PBD) expression construct was provided by Dr. Sheau-Yann Shieh (Academia Sinica, Institute of Biomedical Sciences). The recombinant chemokines CCL21, CXCL12, CXCL10, CCL5 and CCL20 were purchased from PeproTech (London, UK). Piceatannol (P0453) was purchased from Sigma-Aldrich (St. Louis, MO).

### CBAP Knockdown in Jurkat T cells

Recombinant lentivirus expressing CBAP-specific short hairpin RNA (shRNA; target sequence: 5′-ACCGAACGGAGAGCGAAGAAA-3′) was provided by the National RNAi Core Facility at Academia Sinica, Taiwan. Jurkat T cells infected with this recombinant lentivirus were selected in medium containing 3 µg/ml puromycin. Specific clones of interest were later analyzed in medium without puromycin.

### Flow Cytometry

Isolated single-cell suspensions of splenocytes or lymphocytes were pre-incubated with Fcγ-blocking antibody (clone 2.4G2) and then stained with fluorochrome-conjugated antibodies (eBioscience, San Diego, CA) as indicated in each figure. All flow cytometric experiments were performed on a FACSCanto flow cytometer (BD Biosciences, San Jose, CA) and analyzed with FlowJo software (TreeStar, Ashland, OR).

### Antibodies

Antibodies against the following were used in this study: Rac-1 (23A8), Vav1 (05-219), and integrin β1 (AB1952) from Upstate; Vav1 (GTX103489), phospho-Tyr174-Vav1 (GTX61721), ZAP70 (GTX18371 and GTX61094), green fluorescent protein (GFP) (GTX113617), and Myc (GTX20032) from GeneTex (Irvine, CA); phospho-Tyr492-Zap70 (2215-1) and integrin β1 (1798-1) from Epitomics (Burlingame, CA); GFP (632381) from Clontech (Mountain View, CA); integrin β1 (610468) from BD Biosciences (San Jose, CA); talin (T3287) from Sigma-Aldrich (San Louis, MO); CC chemokine receptor (CCR7) (4B12), CXCR4 (2B11), CD62L (MEL-14), and CD3e (145-2C11) from eBioscience (San Diego, CA); integrin β1 (HMβ1-1, TS2/16), integrin β2 (M18/2, TS1/18), CCR5 (HM-CCR5), CCR6 (29-2L17), CXCR3 (CXCR3-173), and CXCR4 (12G5) from BioLegend (San Diego, CA); integrin β1 (HUTS-4) from Millipore (Billerica, MA); and integrin β2 (KIM127) was kindly provided by Dr. Minsoo Kim, University of Rochester. The mouse monoclonal antibody against human CBAP was generated as described [27]. Polyclonal anti-mouse CBAP was generated using bacterially produced recombinant full-length GST-tagged CBAP (Geness Biotech, Taipei, Taiwan).

### *In vivo* Homing Assays

The *in vivo* homing assay was carried out essentially as described [28]. Briefly, LN T cells (6×10^6^) purified from 6- to 8-week-old *CBAP* ^+/+^ or *CBAP* ^−/−^ mice (thy1.2^+^) were injected into the tail vein of thy1.1^+^ recipient C57BL/6 mice. One hour after injection, the recovered thy1.2^+^ CD4^+^ and CD8^+^ T cells in the specified organs were determined by flow cytometry. The number of recovered T cells was taken as homing efficiency. The homing efficiency of *CBAP* ^−/−^ T cells is presented as a relative ratio to that of *CBAP* ^+/+^ T cells, which was set as 1. Alternatively, purified LN T cells from *CBAP* ^+/+^ or *CBAP* ^−/−^ mice were differentially labeled with 0.1 µM CellTracker Green 5-chloromethylfluorescein diacetate (CMFDA; Invitrogen, Carlsbad, CA) and 2 µM PKH26 (Sigma-Aldrich, San Louis, MO), respectively. An equal number of labeled *CBAP* ^+/+^ and *CBAP* ^−/−^ T cells (4×10^6^ each) was mixed and injected intravenously into WT recipient mice. One hour after injection, homing of dye-labeled cells to specified organs was analyzed by flow cytometry, and the relative homing efficiency of *CBAP* ^−/−^ T cells was determined as described above.

### Chemotaxis Assay

*In vitro* chemotaxis assays [13] were performed using a Transwell chamber with a 6.5-mm diameter, 5-µm pore size polycarbonate membrane according to the manufacturer’s instructions (Costar, Tewksbury, MA). Briefly, 1×10^6^ cells in 100 µl of chemotaxis medium (RPMI 1640, 1% bovine serum albumin, 20 mM HEPES [*N*-2-hydroxyethylpiperazine-*N*’-2-ethanesulfonic acid] and 2 mM glutamine) were added to the upper chamber. Specific chemokines were placed in the lower chamber. After incubation for 3 h (primary T cells) or 1 h (Jurkat T cells), numbers of cells in the bottom chamber were determined by an automatic cell counter (Countess, Invitrogen, Carlsbad, CA).

### Static Cell Adhesion Assay

Assays of cell adhesion to intercellular adhesion molecule (ICAM)-1 or vascular cell adhesion molecule (VCAM)-1 were carried out with the Cytoselect Leukocyte-Endothelium Adhesion Assay kit (Cell Biolabs, San Diego, CA; Catalog number CBA-211) with minor modifications. Briefly, 96-well plates (Nalge Nunc, Roskilde, Denmark) were coated with ICAM-1-immunoglobulin crystallizable fragment (Fc) or VCAM-1-Fc (3 µg/ml; R&D Systems, Minneapolis, MN) followed by blocking with PBS containing 2.5% BSA. Cells (2×10^5^) labeled with fluorescent LeukoTracker (Cell Biolabs, San Diego, CA) were incubated with CXCL12 (100 ng/ml) or CCL21 (100 ng/ml) and applied to plates at 37°C for 2 min (Jurkat T cells) or 30 min (primary T cells). After washing, adhered cells were lysed, and fluorescence was quantified with a fluorescence plate reader. The number of adherent cells was determined on the basis of a standard curve obtained with a known number of cells.

### Affinity-based Precipitation of Activated/GTP-bound Rac1 or Cdc42

The active/GTP-bound form of Rac1 (or Cdc42) was detected by affinity-based precipitation as described [29]. Briefly, lysates from unstimulated cells or cells stimulated with a chemokine were incubated at 4°C for 1 h with rotation (25 rpm) with 10 µg of purified GST-PBD beads. After washing, the pulled-down precipitates were separated by sodium dodecyl sulfate-polyacrylamide gel electrophoresis (SDS-PAGE) and analyzed by immunoblotting with monoclonal anti-Rac1 (or anti-Cdc42).

### *In vitro* T-cell Receptor–mediated Activation of Primary T cells

Primary T cells were purified from LNs of mice by a pan-T cell marker CD90.2^+^, also named thy1.2, magnetic cell sorter (Miltenyi Biotec, Bergisch Gladbach, Germany) according to the manufacturer’s instructions. Cells were immediately cultured in a dish coated with anti-CD3 (5 µg/ml) and anti-CD28 (2.5 µg/ml) for 72 h.

### Statistical Analyses

Statistical comparisons between all data sets were performed with a nonparametric 2-tailed Mann-Whitney test implemented in GraphPad InStat (GraphPad Software, La Jolla, CA). Significant differences are indicated in the figures (*P<0.05, **P<0.01, and ***P<0.005). Data displaying statistical analyses were performed at least three times.

## Results

### CBAP is Required for Optimal Chemokine-induced T-cell Migration and Adhesion

**Figure 1:**
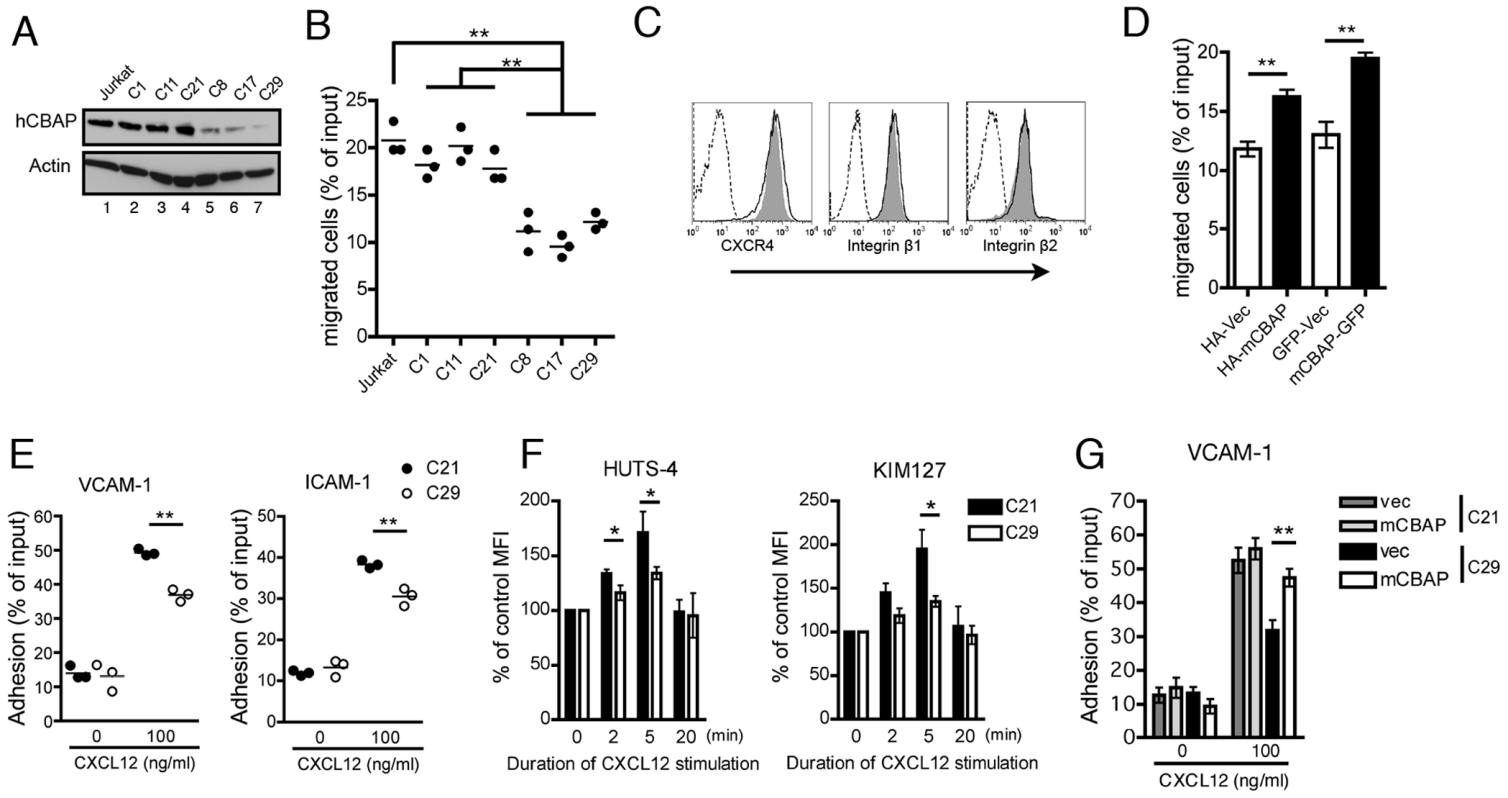
CBAP is required for CXCL12-induced migration and adhesion and activation of integrins in Jurkat T cells. (A) Total cell lysates from the parental and six independent CBAP-shRNA-transfected Jurkat clones, as indicated, were immunoblotted with antibodies against human CBAP and β-actin. (B) Transwell chemotaxis of the parental and CBAP-shRNA knockdown clones was measured in the presence of CXCL12 (100 ng/ml) (each dot represents a duplicated measurement). (C) Expression of surface CXCR4, integrin β1, and integrin β2 on control C21 (gray shading) and CBAP-knockdown C29 cells (black line) was measured by flow cytometry. Data are representative of two independent experiments. (D) Rescue of the CXCL12-dependent migration defect in C29 cells. Migration of cells ectopically expressing HA-tagged or GFP-tagged murine CBAP was measured with a Transwell assay (three repeats of duplicated experiments). (E) Adhesion of control C21 cells and C29 cells to wells coated with VCAM-1 or ICAM-1 in the presence of CXCL12 (100 ng/ml). Adhesion is presented as the percent of input cells that were retained inside the well after rinsing with medium. N = 3. (F) Activation of integrin determined by staining with an integrin-specific monoclonal antibody directed against the active conformation of integrin α4β1 (HUTS-4) or integrin αLβ2 (KIM127). Jurkat cells stimulated with CXCL12 were labeled with the indicated antibodies and subjected to flow cytometry. Data are presented as the percentage of mean fluorescent intensity (MFI) compared with control unstimulated cells. N = 3. (G) Integrin α4β1–mediated adhesion of Jurkat clones (C21 and C29) expressing GFP-tagged murine CBAP in the absence or presence of CXCL12. N = 3. Data shown above are mean ± SD from independent experiments, as indicated, in duplicate. *P<0.05 and **P<0.01.

CBAP was first identified as a binding protein of the βc subunit in hematopoietic cells [27], whereas integrin β1 was found to associate with βc in endothelial cells and plays an important role during vasculogenesis and tumor angiogenesis [25], [26], leading us to hypothesize that CBAP may also be involved in integrin-related cellular function, such as cell adhesion and migration. To this end, we generated several stable human Jurkat T-cell clones in which CBAP expression was decreased using a lentiviral vector encoding a CBAP-specific shRNA (see Materials and Methods) and tested the effect of reducing CBAP levels on T cell adhesion and migration induced by chemokine CXCL12, which is the ligand for CXCR4, the major chemokine receptor in Jurkat T cells. We first found that three stable clones (C8, C17, C29) with successful knockdown of CBAP expression (Figure 1A, lanes 5–7) had a profoundly lower response to CXCL12-induced migration (Figure 1B, columns C8, C17, C29) compared to the parental Jurkat T cells or the other unsuccessful CBAP-knockdown cell lines (C1, C11, and C21) (Figure 1A, lanes 2–4; Figure 1B, columns C1, C11, C21). Chemokine receptor CXCR4, integrin β1, and integrin β2 on the cell surface were expressed similarly between these control and knockdown clones (Figure 1C), excluding the possibility that CBAP knockdown affected the expression of those gene products. Moreover, expression of HA-tagged or GFP-tagged mouse CBAP (mCBAP-GFP), which was resistant to human CBAP shRNA–dependent downregulation, efficiently rescued the migration defect of CBAP-knockdown Jurkat C29 cells (Figure 1D). These results further supported that CBAP acts as a positive regulator in chemokine-dependent T-cell migration.

To examine whether CBAP is also involved in chemokine-dependent T-cell adhesion, we performed a static adhesion assay. We found that CXCL12-induced adhesion to plates coated with VCAM-1 or ICAM-1 (ligand for integrins α4β1 and αLβ2, respectively) increased profoundly in control C21 cells (Figure 1E, solid bars). However, CBAP-knockdown C29 cells exhibited attenuated static adhesion under the same conditions (Figure 1E, open bars). This is consistent with the observation that, after CXCL12 treatment and compared to control C21 cells, C29 cells displayed significantly reduced binding of an integrin-specific monoclonal antibody directed against the active conformation of integrin α4β1 (HUTS-4) or integrin αLβ2 (KIM127) (Figure 1F). Expression of murine CBAP proteins also significantly rescued the adhesion defect of C29 cells following CXCL12 stimulation (Figure 1G), suggesting that CBAP is involved in chemokine-induced cell migration and integrin-mediated adhesion of T cells. Overexpression of CBAP in C21 Jurkat cells did not further increase adhesion (Figure 1G), suggesting that CBAP-mediated integrin-dependent adhesion is already saturated.

**Figure 2:**
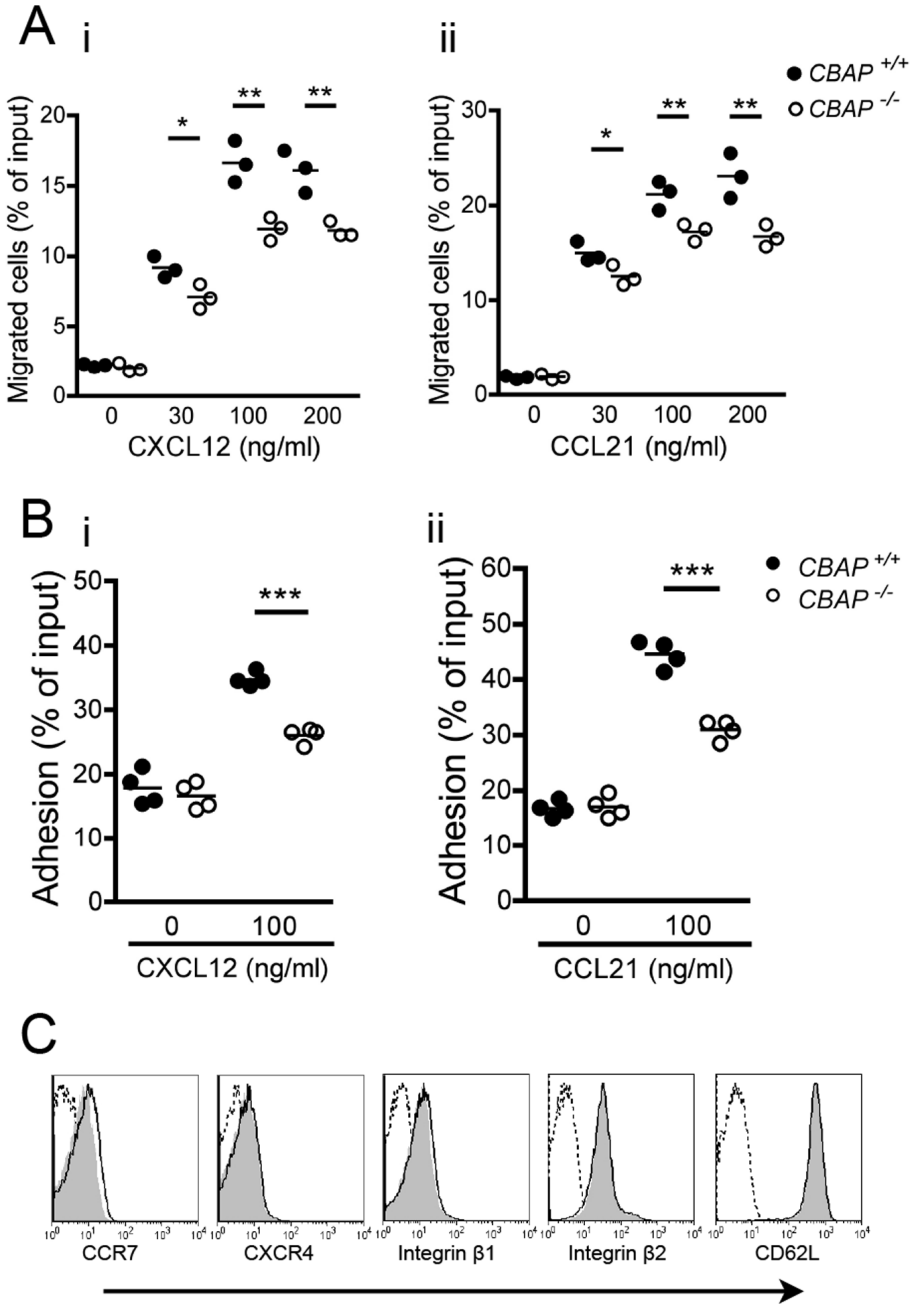
*CBAP^−/−^* primary T cells display reduced chemokine-dependent cell migration and adhesion *in vitro*. (A) Chemotaxis of purified LN T cells from *CBAP* ^+/+^ and *CBAP* ^−/−^ mice in response to different doses of CXCL12 (Ai) or CCL21 (Aii). Data shown are from three independent experiments in duplicate. (B) Adhesion of LN T cells to immobilized VCAM-1 in the presence of CXCL12 (Bi) or CCL21 (Bii). Data represent the mean of four independent experiments in duplicate. (C) Expression of surface CXCR4, CCR7, CD29, CD18 and CD62L on LN T cells (CD90.2^+^) from *CBAP* ^+/+^ (gray shading) and *CBAP* ^−/−^ (black line) mice was determined by flow cytometry. Isotype control is shown as a dashed line. Data shown are representative of three independent experiments. *P<0.05, **P<0.01 and ***P<0.005.

To elucidate the function of CBAP in primary T cells, we generated CBAP-deficient (*CBAP* ^−/−^) mice by a conventional gene knockout strategy, which resulted in complete deletion of *CBAP* (Figure S1A & S1B). *CBAP* ^−/−^ mice lacked expression of both CBAP mRNA and protein in liver (Figure S1C & S1D). Owing to very low CBAP protein expression in mouse LNs (below the detection limit of our anti-mCBAP), we used RT-PCR to confirm that CBAP mRNA was expressed in WT but not *CBAP* ^−/−^ T cells (Figure S1C, lanes 5 and 7). To further study the novel function of CBAP in T-cell trafficking, purified LN T cells were stimulated *in vitro* with CXCL12 or CCL21, two important chemokines for interstitial migration within LNs. Using an *in vitro* chemotaxis assay in Transwells, migration of WT T cells toward each chemokine increased in a dose-dependent manner with a peak at 100 ng/ml for CXCL12 (Figure 2Ai). A similar migration defect was observed in the CCL21-driven chemotactic assay of *CBAP* ^−/−^ T cells compared with that of WT counterparts (Figure 2Aii). Moreover, we found that *CBAP* ^−/−^ T cells exhibited defective static adhesion in response to CXCL12 and CCL21 stimulation compared with WT cells (Figure 2Bi and 2Bii). Further analysis indicated that this migration and adhesion defect was not due to reduced surface expression of chemokine receptors CXCR4 and CCR7, integrins β1 and β2, or L-selectin CD62L on *CBAP* ^−/−^ LN T cells (Figure 2C). These results further supported that CBAP may be directly involved in chemokine-induced migration and adhesion of primary T-cells.

**Figure 3:**
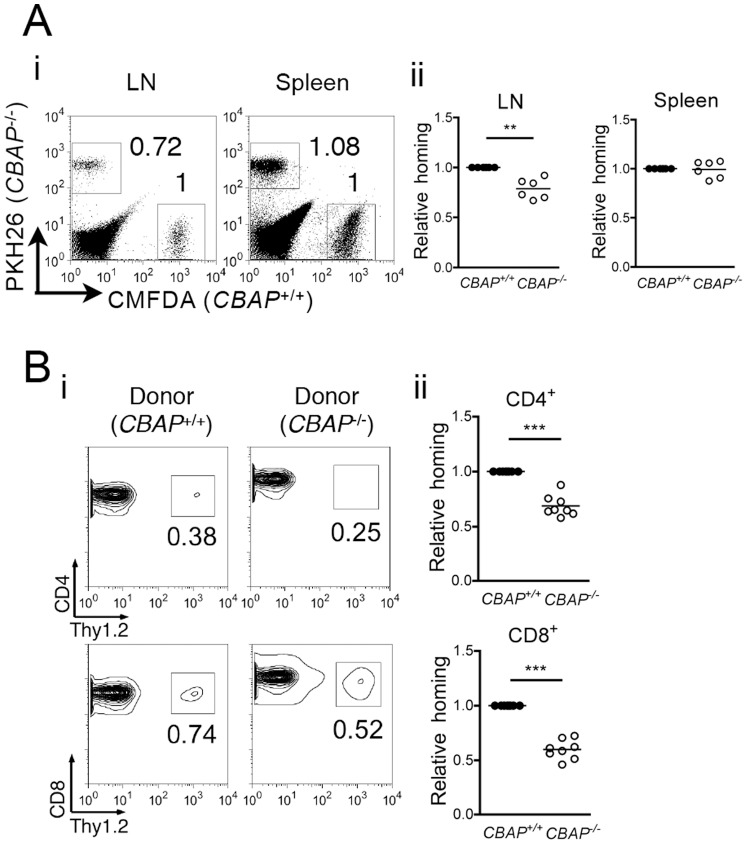
CBAP is critical for T-cell homing to LNs. (Ai) Representative flow cytometry profiles of labeled T cells from *CBAP* ^+/+^ and *CBAP* ^−/−^ mice 1 h after adoptive transfer. T cells from *CBAP* ^+/+^ and *CBAP* ^−/−^ mice were differentially labeled with CMFDA and PKH26, respectively. The ratio of the recovered *CBAP* ^−/−^ cells to recovered *CBAP* ^+/+^ T cells is shown beside each boxed area in the indicated tissues (see Materials and Methods). (Aii) Differential homing efficiency of *CBAP* ^+/+^ and *CBAP* ^−/−^ T cells. N = 6. (Bi) Representative flow cytometric profiles of thy1.2^+^/CD4^+^ and thy1.2^+^/CD8^+^ T cells in thy1.1^+^ (WT) recipient LNs 1 h after adoptive transfer (see Materials and Methods). Numbers beside the boxed areas represent the percentage of donor thy1.2^+^ T cells within total CD4^+^ (upper panel) or CD8^+^ (lower panel) T-cell populations. (Bii) Relative efficiency of *CBAP* ^−/−^ CD4^+^ and CD8^+^ T cell homing to LNs. N = 8. Data shown are mean ± SD from independent experiments, as indicated, in duplicate. **P<0.01 and ***P<0.005.

Campbell et al. [30] demonstrated that chemokine signaling is essential for firm adhesion of rolling T cells in high endothelial venules before entering LNs. Because CBAP deficiency impaired chemokine-dependent T-cell mobility and adhesion *in vitro*, we reasoned that CBAP might play a role in T-cell trafficking *in vivo*. We therefore performed an *in vivo* T-cell homing assay by adoptive transfer of dye-labeled T cells and measured donor cell recovery in LNs 1 h after transplantation (see Materials and Methods). The results showed that the relative homing efficiency of *CBAP* ^−/−^ donor T cells into LNs was indeed reduced (∼24%) compared to that of control counterparts (Figure 3Ai and Aii). Of note, the homing efficiency of *CBAP* ^−/−^ T cells into spleen was comparable to that of control donors (Figure 3Ai and Aii). Similar results were observed when the homing assay was conducted by transferring control or *CBAP* ^−/−^ donor T cells (Thy1.2^+^) into C57BL/6J-Thy1.1 recipient mice (see Materials and Methods), i.e., the homing efficiency of *CBAP* ^−/−^ CD4^+^ and CD8^+^ T cells into LNs (Figure 3Bi) was approximately 73% and 65%, respectively, of the control counterparts (Figure 3Bii), further confirming the defect of *CBAP* ^−/−^ T cells in LN homing.

### Altered Chemokine-activated Signaling in *CBAP* ^−/−^ T cells

**Figure 4:**
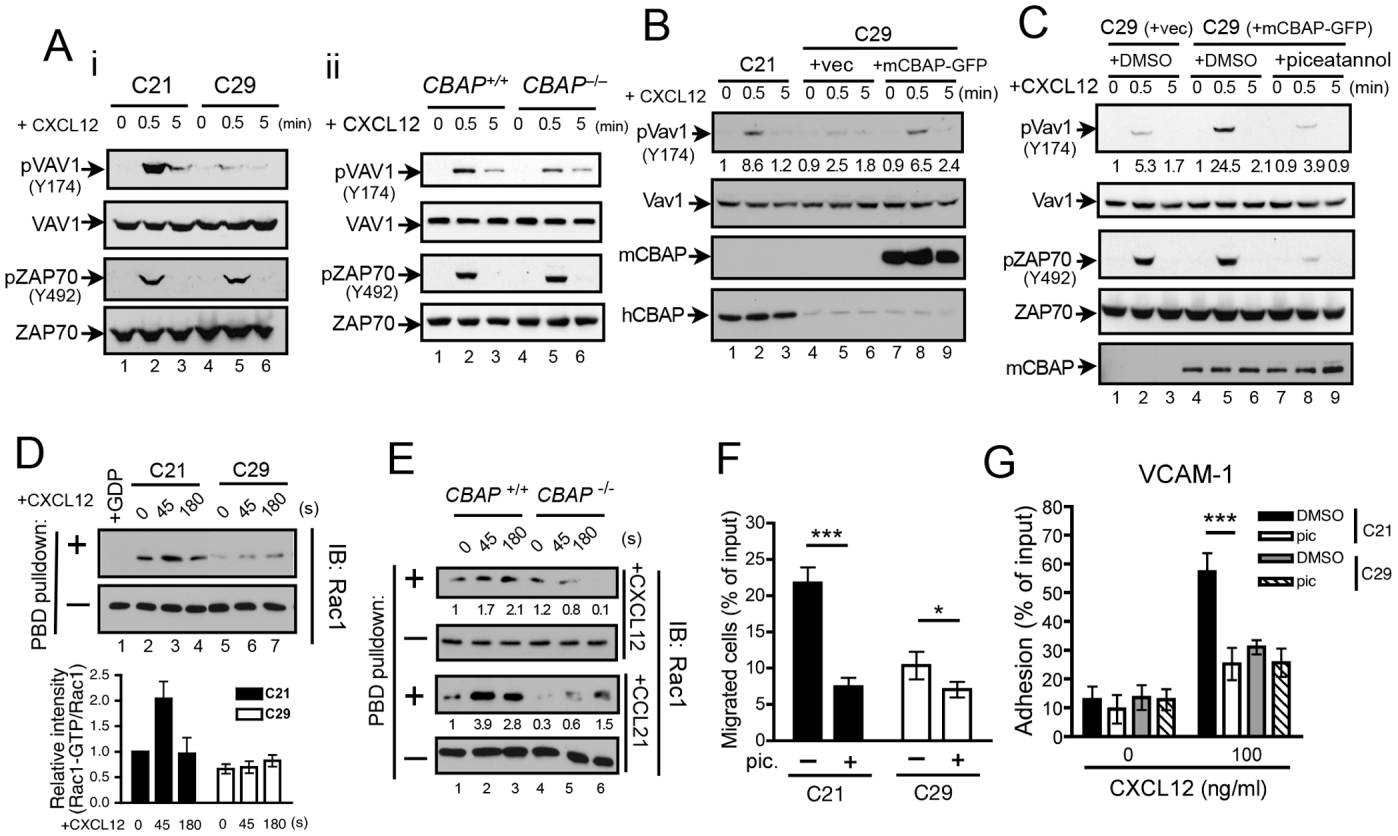
CBAP is important for chemokine-dependent activation of Vav1 and Rac1 in T cells. Analysis of chemokine-induced activation of ZAP70 and Vav1 in (Ai) Jurkat T cells and (Aii) primary T cells. Cells were stimulated with CXCL12 (200 ng/ml) at the indicated times. Total cell lysates were subjected to immunoblotting with antibodies as indicated. (B) CBAP-knockdown (C29) cells transfected with a plasmid encoding enhanced GFP (vec) or GFP-tagged murine CBAP (mCBAP-GFP) were stimulated with CXCL12 and subjected to immunoblotting with antibodies as indicated. (C) Effect of piceatannol treatment on CBAP-dependent Vav1 phosphorylation. After a 3-h incubation with DMSO or piceatannol (25 µM), cells were stimulated with CXCL12 and subjected to immunoblotting with antibodies as indicated. The numbers indicated below the pVav1 blots are presented as the fold change over the control bands (B, C). (D) CXCL12-induced activation of Rac1 was analyzed by PBD-pulldown assay (see Materials and Methods). C21 and C29 Jurkat T-cell clones were stimulated with CXCL12 for the indicated times. Total cell lysates were prepared and pulled down with GST-PBD and subjected to immunoblotting to detect Rac1 (upper panel). Each total cell lysate was also blotted directly with anti-Rac1 as a loading control (lower panel). Quantified data are shown below the blot as relative intensity of Rac1-GTP compared to that at the zero time point from the same samples of three independent experiments. (E) Activation of Rac1 in *CBAP* ^−/−^ primary T cells was analyzed after stimulation with CXCL12 (200 ng/ml) or CCL21 (200 ng/ml). *CBAP* ^+/+^ and *CBAP* ^−/−^ T cells purified from LNs were stimulated with CXCL12 or CCL21 for the indicated times. Total cell lysates were analyzed, and the activity of Rac1-GTP is presented as described in (D). (F, G) Effect of piceatannol treatment on the migration and adhesion of CBAP-knockdown Jurkat clones. DMSO- or piceatannol (pic)-pretreated cells were stimulated with CXCL12 and analyzed for cell migration (F) and adhesion (G) as described in the legends to Figure 1B and 1E, respectively. The data presented represent the mean ± SD of five individual experiments. *P<0.05 and ***P<0.005.

Stimulation of chemokine receptors activates signal transduction through trimeric G protein complexes and multiple downstream pathways, including activation of phospholipase Cβ, leading to an increase in intracellular concentrations of Ca^2+^ and activation of PI3Kγ and its downstream kinase Akt [31]. We found that CXCL12- or CCL21-induced PI3K-dependent phosphorylation of Akt was normal in *CBAP* ^−/−^ T cells and that Ca^2+^ was mobilized to a similar extent in both control (i.e., C11 and C21) and CBAP-knockdown (i.e., C17 and C29) Jurkat T-cell clones (data not shown). In a leukemia cell line and human peripheral blood, CXCL12-induced tyrosine phosphorylation of ZAP70 and Vav1 is essential for cell migration [8], [32] and adhesion [10], [33], [34]. We thus examined the possibility that CBAP is involved in CXCL12-induced activation of the ZAP70/Vav1/Rac1 signaling axis. As shown in Figure 4Ai and 4Aii (lower panels), knockdown or knockout of CBAP did not significantly affect CXCL12-induced phosphorylation of ZAP70 at Y492, which stands for major activation site for ZAP70 [35]. However, it markedly reduced the phosphorylation of Vav1 at Y174, which is a key phosphorylation site of Vav1 as an indicator of GEF activity [36]. (Figure 4Ai and 4Aii, upper panels, compare lanes 2 and 5). Furthermore, ectopic expression of mCBAP-GFP in C29 cells efficiently restored CXCL12-induced phosphorylation of Vav1, but it did not affect phosphorylation of ZAP70 (Figure 4B, compare lanes 5 and 8; and Figure 4C, compare lanes 2 and 5). Treatment with the ZAP70 kinase inhibitor, piceatannol, significantly suppressed ZAP70 phosphorylation at Y492 and inhibited CBAP-dependent Vav1 phosphorylation (Figure 4C, compare lanes 5 and 8). These data suggested that CBAP is essential for ZAP70-dependent phosphorylation of Vav1. The Rho-family GTPases, such as Rac1 and Cdc42, control cell migration and adhesion by modulating the rearrangement of the actin cytoskeleton [37] and working downstream of Vav1. To investigate whether reduced Vav1 phosphorylation in CBAP-knockdown T cells would result in reduced Rho GTPase activity, we performed affinity-based pulldown assays using GST-PBD to determine the amount of active (i.e., GTP-bound) Rac1 and Cdc42. Upon CXCL12 stimulation, the level of GTP-bound Rac1 increased significantly within 45 s in control C21 T cells (Figure 4D, lane 3), but under the same conditions there was no significant induction of Rac1-GTP in C29 cells (Figure 4D, lanes 6, 7). A similar reduction in the level of GTP-bound Cdc42 was also observed in CBAP-knockdown cells (data not shown). Attenuated activation of Rac1 was also apparent in primary *CBAP* ^−/−^ T cells in response to CXCL12 or CCL21 stimulation (Figure 4E). To further investigate whether both CBAP and ZAP70 work in the same signaling pathway to regulate T-cell adhesion and migration, *in vitro* cell migration and adhesion analyses were carried out with CBAP-knockdown cells in the absence or presence of piceatannol. Indeed, piceatannol markedly inhibited CXCL12-mediated migration or VCAM-1-mediated adhesion of C21 cells. However, it had marginal or no effect on the migration or adhesion of C29 cells (Figure 4F & Figure 4G), suggesting that CBAP mainly works in the ZAP70-dependent pathway. Together, these results suggested that CBAP is an important component in ZAP70-dependent activation of the Vav1/Rac1 signaling axis.

### CBAP pre-associates with Integrin β1 and ZAP70 and Facilitates Chemokine-Induced ZAP70-mediated Dissociation of the Vav1-talin Complex

**Figure 5:**
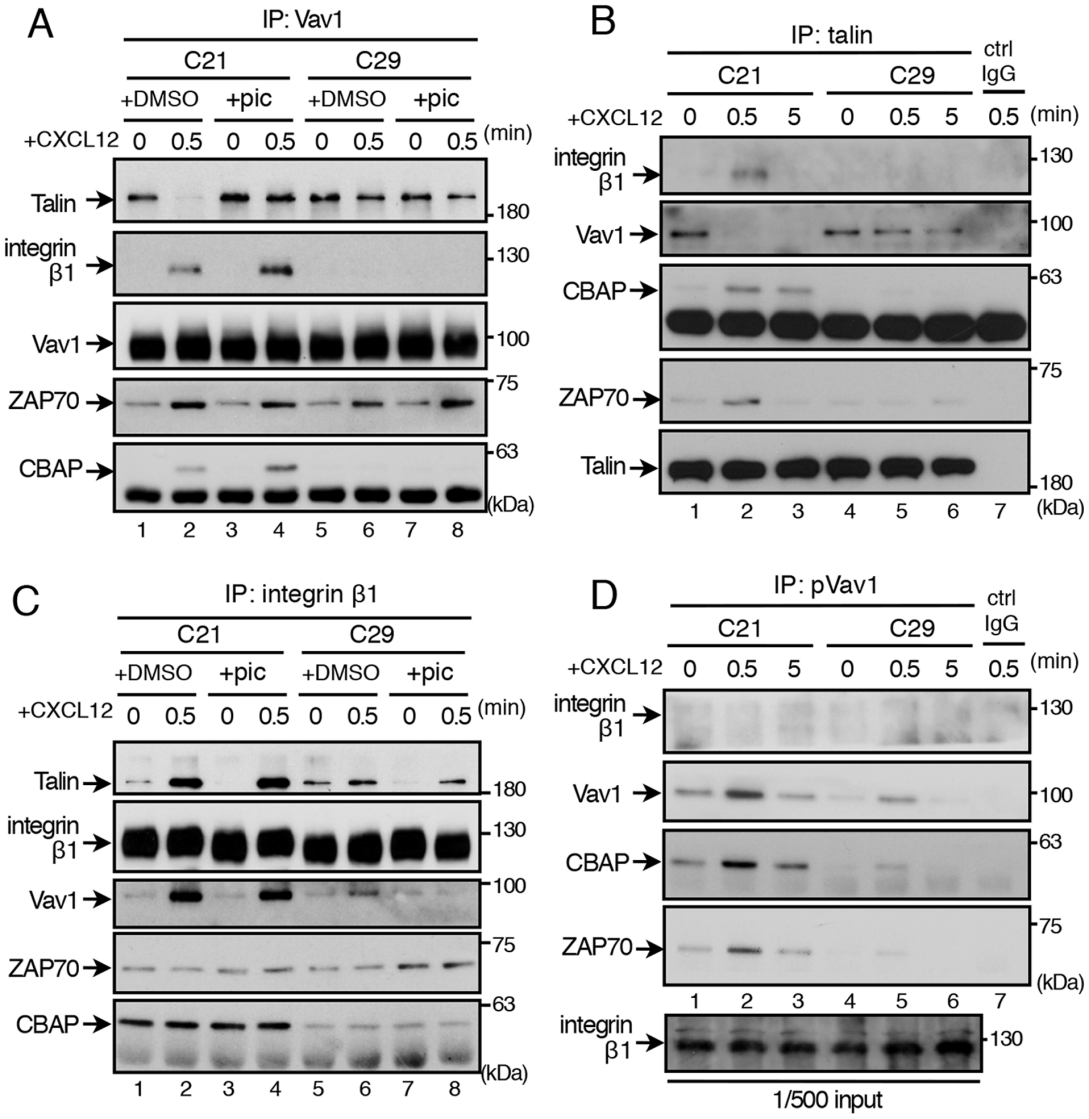
CBAP pre-associates with integrin β1 and ZAP70 and facilitates chemokine-induced ZAP70-mediated dissociation of the Vav1-talin complex. (A) Control (C21) or CBAP-knockdown cells (C29) were stimulated with CXCL12 (200 ng/ml) at the indicated times. Lysates from cells pretreated with DMSO or piceatannol (25 µM) were subjected to immunoprecipitation (IP) with anti-Vav1 and immunoblotting with the indicated antibodies. (B) CXCL12-stimulated cells as described in (A) were lysed and immunoprecipitated with anti-talin or control (ctrl) IgG and subjected to immunoblotting with the indicated antibodies. (C) Same as in (A) except that cell lysates were immunoprecipitated with anti-integrin β1 followed by immunoblotting with the indicated antibodies. (D) Same as in (A) except that cell lysates were immunoprecipitated with anti-pVav1 before immunoblotting with the indicated antibodies. Each of panels A–D presents one representative result from three independent experiments that yielded very similar results.

During the process of inside-out signaling of chemokine receptors, chemokine-induced ZAP70-mediated dissociation of the Vav1-talin complex is required to generate high-affinity integrin α4β1 conformations [10], [33], [34]. We next examined whether CBAP plays a role in chemokine-induced dissociation of the Vav1-talin complex. To test this possibility, cell lysates from control (C21) or CBAP-knockdown cells (C29) without or with CXCL12 stimulation were immunoprecipitated with anti-Vav1 or anti-talin, and co-precipitated molecules were analyzed. An obvious complex between Vav1 and talin was detected with both anti-Vav1 and anti-talin before CXCL12 stimulation, but this complex disappeared rapidly after chemokine treatment (Figure 5A lanes 1, 2, and 5B lanes 1–3). Significantly, CBAP knockdown in Jurkat T cells (C29) abolished the dissociation of the Vav1-talin complex (Figure 5A, lanes 5, 6, and 5B, lanes 4–6), albeit CXCL12-induced ZAP70 activation could still be detected (Figure 4Ai, lane 5). Moreover, CXCL12 stimulation enhanced the formation of a complex containing integrin β1, Vav1, ZAP70 and CBAP that could be immunoprecipitated by anti-Vav1, and this complex was not detected in C29 cells, albeit in the latter cells ZAP70 could still be co-immunoprecipitated by anti-Vav1 (Figure 5A, compare lanes 2 and 6). Likewise, CXCL12 stimulated the formation of a complex containing talin, integrin β1, ZAP70 and CBAP that could be immunoprecipitated by anti-talin (Figure 5B, lanes 1, 2), and formation of this complex was not detected in C29 cells (Figure 5B, compare lanes 2 and 5).

**Figure 6:**
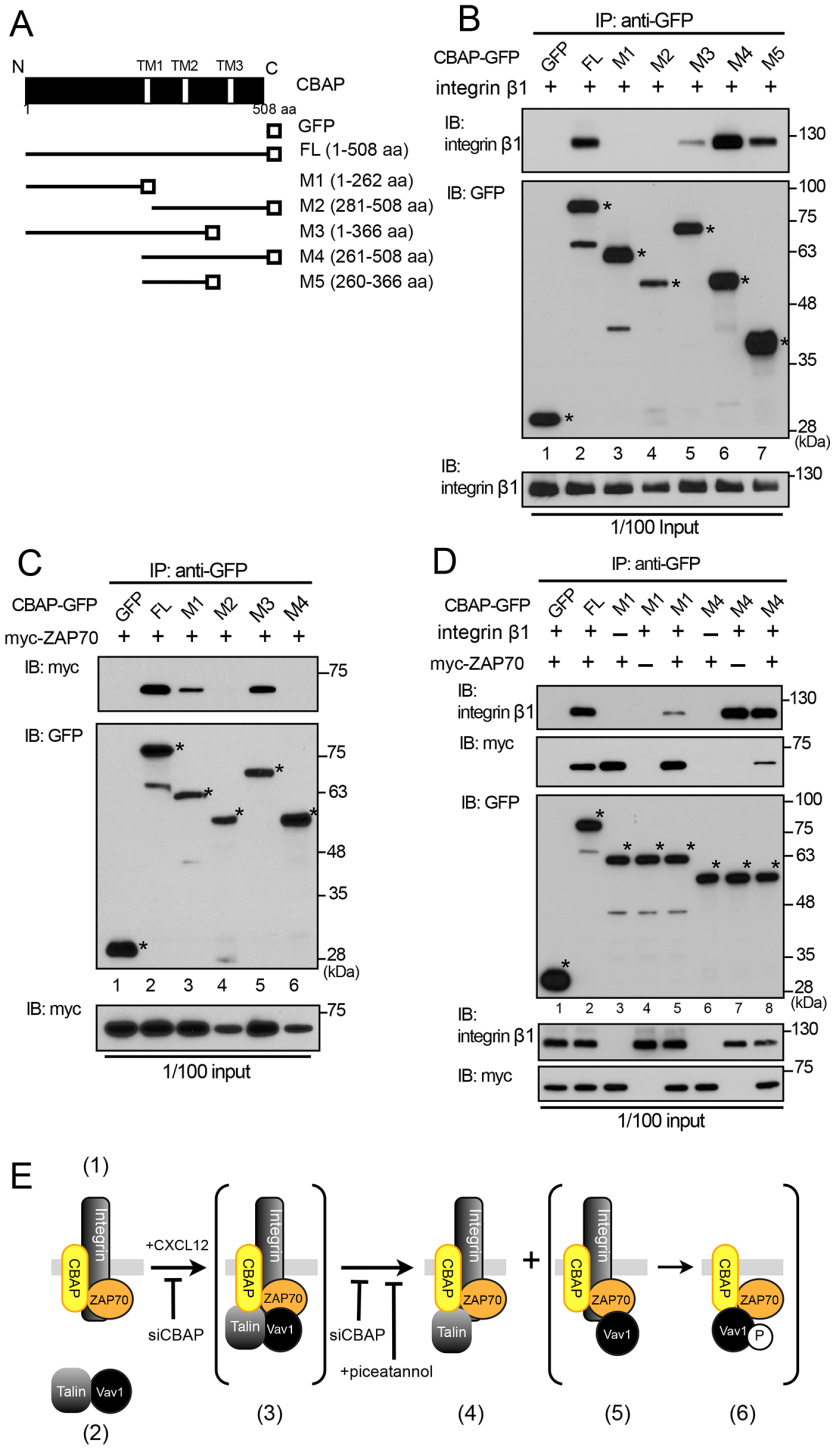
Complex formation among CBAP, ZAP70 and integrin β1. (A) Schematic representation of GFP-tagged full-length (FL) and various deletion mutants (M1–M5) of CBAP. aa, amino acid. (B, C, D) Cell lysates from 293T cells co-expressing various combinations of the indicated proteins were immunoprecipitated (IP) with anti-GFP followed by immunoblotting (IB) using the indicated antibodies. As input controls, total cell lysates (1/100) were also blotted with the relevant antibody, and the results are shown at the bottom of each panel. (E) A proposed model for the role of CBAP in chemokine-induced ZAP70-mediated dissociation of the Vav1-talin complex (see text for details). Components in Black-and-white have been established previously [10], and Components in color were established in the current study.

Next, we noticed that similar amounts of ZAP70 and CBAP were detected in the anti-integrin β1 immune complexes when control C21 cells were left unstimulated or stimulated with chemokine (Figure 5C, compare lanes 1 and 2), suggesting that integrin β1, ZAP70, and CBAP constitutively associate in T cells. This complex could also be detected in a non-T-cell system ectopically overexpressing these three molecules (see text for Figure 6 below), and treatment with piceatannol did not affect the formation of this complex (Figure 5C, lanes 3, 4). Notably, CXCL12 treatment increased the amount of talin and Vav1 detected in the anti-integrin β1 immune complex (Figure 5C, compare lanes 1 and 2). On the other hand, consistent with earlier studies [10], [33], [34], piceatannol treatment abolished CXCL12-induced dissociation of the talin/Vav1 complex (Figure 5A, lane 4). Notably, under such conditions, talin was detected in the immune complex precipitated by anti-Vav1, which contains integrin β1, Vav1, ZAP70 and CBAP (Figure 5A, top panel, lane 4). Last, CBAP and ZAP70 but not integrin β1 were detected in the complex precipitated by anti-pVav1 from CXCL12-treated control (C21) cell lysates (Figure 5D, lane 2), and formation of this complex (pVav1/CBAP/ZAP70) was not induced by CXCL12 in C29 cells (Figure 5D, lane 5).

Next, we examined the spatial interactions between integrin β1, ZAP70 and CBAP that were ectopically overexpressed in a non-T-cell line (human 293T cells), which expressed no detectable level of endogenous integrin β1 or ZAP70. In this experiment, 293T cells overexpressing GFP-tagged full-length CBAP (Figure 6A; FL) or various deletion mutants of CBAP (Figure 6A; M1 to M5) along with integrin β1 and/or myc-tagged ZAP70 were lysed and immunoprecipitated with anti-GFP followed by immunoblotting with anti-integrin β1 or anti-myc (Figure 6B–D). The results indicated that all three putative transmembrane domains (TM1–3) located at the C-terminal half of CBAP are required for stable association of CBAP with integrin β1, as loss of TM1 (compare M2 and M4 mutants, Figure 6B) or TM3 (compare M4 and M5, Figure 6B, lanes 6 and 7) markedly attenuated the association. On the other hand, the N-terminal but not the C-terminal domain of CBAP was found to be critical for CBAP interaction with ZAP70 (compare M1 and M3 to M2 and M4, Figure 6C). Furthermore, within the ZAP70/CBAP/integrin β1 complex, integrin β1 and ZAP70 apparently have direct contact with each other, as integrin β1 could be co-immunoprecipitated with the M1 mutant only when myc-ZAP70 was co-expressed (Figure 6D, compare lanes 3–5) and myc-ZAP70 could be co-immunoprecipitated with the M4 mutant only when integrin β1 was co-expressed (Figure 6D, compare lanes 6–8). These results, together with those of Figure 5, suggested that upon chemokine stimulation the Vav1-talin complex, as a whole, transiently binds to the preformed integrin β1/CBAP/ZAP70 complex, thereby allowing ZAP70 to phosphorylate Vav1 and dissociate the Vav1-talin complex (Figure 6E).

### Impaired Chemotaxis in *CBAP^−/−^* Activated Primary T cells

**Figure 7:**
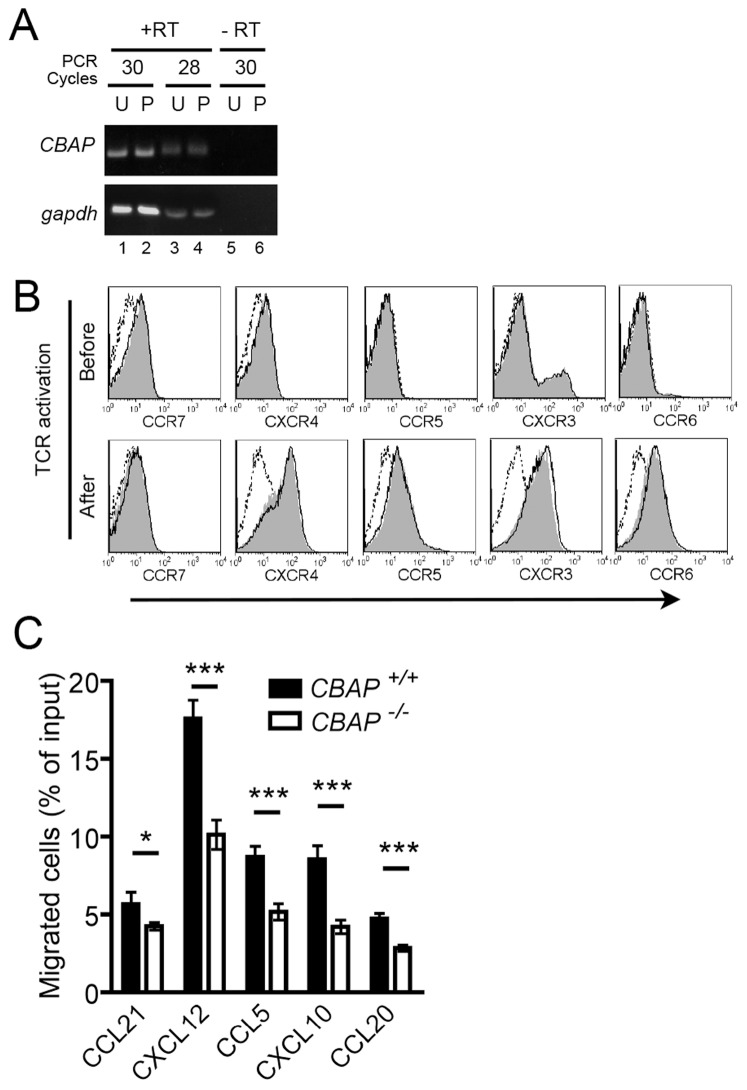
*CBAP* ^−/−^ activated T cells display reduced chemotaxis toward each of several chemokines. (A) Semi-quantitative RT-PCR analysis reveals CBAP mRNA expression from unprimed (U) purified CD90.2^+^ LN T cells or primed (P) T cells activated for 72 h with plate-bound anti-CD3/CD28. Glyceraldehyde 3-phosphate dehydrogenase (gapdh) mRNA expression is shown as an internal control. Data are representative of three independent experiments. The minus-reverse transcriptase (-RT) controls were indicative of contaminating genomic DNA in the samples. (B) Flow cytometry of the expression of chemokine receptors by T-cell receptor (TCR)-activated T cells from *CBAP* ^+/+^ (gray shading) or *CBAP* ^−/−^ (black line). Isotype IgG control is shown as a dashed line in the histogram. Data are representative of three independent experiments. (C) Transwell migration assay of activated T cells from *CBAP* ^+/+^ and *CBAP* ^−/−^ mice in response to the indicated chemokines. Shown are the mean ± SD of migration indices from four independent experiments, each with triplicate samples. *P<0.05 and ***P<0.005.

Our results obtained thus far demonstrated that CBAP regulates the migration of naïve T cells upon stimulation by chemokine CXCL12 and CCL21. To further investigate whether CBAP plays a role in mediating activated T-cell migration, we first checked whether expression of CBAP mRNA is upregulated in activated T cells. By semi-quantitative RT-PCR analysis, the levels of CBAP mRNA were not changed before and after T-cell receptor stimulation (Figure 7A). Then we checked whether the surface expression of inflammatory chemokine receptors and some integrin β subunits in activated T cells changed upon deletion of *CBAP*. Our flow cytometric data showed that the expression of inflammatory chemokine receptors (CXCR4, CCR5, CXCR3 and CCR6) was upregulated in activated WT cells whereas the homeostatic chemokine receptor CCR7 was downregulated; the levels of all these receptors in *CBAP* ^−/−^ T cells were similar to those observed in activated WT cells (Figure 7B). Finally, we used Transwells to analyze the migration of T-cell receptor–activated LN T cells in response to stimulation by any of the four inflammatory chemokines CXCL12, CCL5, CXCL10 or CCL20. As shown in the Figure 7C, *CBAP* **^−/−^** activated T cells displayed significantly reduced migration efficiency toward these inflammatory chemokines compared to WT counterparts. Together, these data suggested that CBAP is expressed not only in naïve T cells but also in activated T cells, where it also plays an important role in controlling chemokine-dependent migration.

## Discussion

The molecular mechanism underlying recruitment of T cells to specific lymphoid organs and inflammatory sites by chemokines–a key step in the communication between innate and adaptive immunity–has been extensively studied but is far from clearly understood. Recently the chemokine-induced ZAP70-mediated activation of Vav1 and integrin signaling was shown to be critical for transmitting chemokine signals to activate adhesion molecules and the migration machinery in T cells [8], [10]–[13], [22]. In this report, we demonstrated that CBAP is a novel component in chemokine-induced ZAP70-mediated signaling event that is involved in T-cell homing to lymphoid organs.

*CBAP* **^−/−^** T cells exhibited moderately decreased migration in response to each of several chemokines, including the homeostatic chemokines CCL21 and CXCL12 (Figure 2) and the inflammatory chemokines CXCL12, CCL5, CXCL10 and CCL20 (Figure 7). However, such a defect was not as dramatic as that observed in mice upon targeted ablation of a specific chemokine receptor, like CCR7 [38], [39] and CXCR4 [39], [40], suggesting that the site of CBAP function may be remote from chemokine receptors and is proximal to integrin complexes and the actin cytoskeleton. ZAP70 was reported to directly interact with the cytoplasmic region of β integrin in a phosphotyrosine-independent manner [41]. Consistent with this result, we observed that CBAP constitutively associated with ZAP70 and integrin β1 (Figure 5C and Figure 6E, complex 1). Garcia-Bernal et al. recently proposed that chemokines initially promote the association of talin/Vav1 and integrin β1 in a ZAP70-independent manner, but thereafter Vav1 is phosphorylated by ZAP70 and quickly dissociates from the Vav1/talin/integrin β1 complex [10]. The newly released talin then further activates integrin α4β1 to yield a high-affinity conformation of this integrin [10]. In this study, we provide evidence that CBAP is a novel component involved in chemokine-induced dissociation of the Vav1/talin complex. Based on our experimental results and those reported by Garcia-Bernal et al. [10], we propose a model for the functional role of CBAP in such a process (Figure 6E). In this model, CBAP constitutively associates with integrin β1 and ZAP70 in T cells (complex 1). Upon chemokine stimulation, the Vav1-talin complex (complex 2) transiently associates with the preformed ZAP70/CBAP/integrin β1 complex to generate complex 3; a particular conformation of this complex enables Vav1 to be phosphorylated by ZAP70. Immediately following the formation of complex 3, it is “transformed” into at least three types of complexes, i.e., complexes 4, 5 and 6, that can be identified with co-immunoprecipitation. Complex 4 contains ZAP70, CBAP, integrin β1 and talin, which renders the activation of integrin α4β1. Complex 5 contains ZAP70, CBAP, integrin β1 and Vav1, which upon phosphorylation of Vav1 by ZAP70 quickly dissociates from integrin β1 and transforms into complex 6 (ZAP70/CBAP/pVav1) that can further activate downstream targets like Rac1. More experiments will be required to test this model.

Although chemokine-induced binding of Vav1-talin to the integrin β1/CBAP/ZAP70 complex is dependent on the presence of CBAP (Figure 5C, compare lanes 2 and 6), we noticed that chemokine treatment still increased the binding of Vav1 to ZAP70 in C29 cells (Figure 5A, lane 6), albeit such treatment did not render Vav1 to be phosphorylated by ZAP70 in these cells. Furthermore, mCBAP overexpression in control C21 cells, expressing abundant endogenous CBAP, could not further sufficiently promote chemokine-induced chemotaxis (data not shown) and adhesion (Figure 1E) *in vitro*. These results suggest that CBAP may serve as an adaptor or scaffold in the integrin β1/CBAP/ZAP70-containing complex that, upon chemokine treatment, would allow Vav1 or ZAP70 (or both) to adopt an optimal conformation(s) for ZAP70 to phosphorylate Vav1. Our preliminary study indicated that CBAP can simultaneously interact with ZAP70 and Vav1. How such interaction facilitates ZAP70-mediated phosphorylation of Vav1 remains to be determined.

A genome-wide proteomic study of the 14-3-3 binding protein family revealed that CBAP (also called TMEM102) is one of many 14-3-3–binding components [42]. 14-3-3 can directly compete with talin for binding to the cytoplasmic tail of integrin β2 [43], [44]. It is possible that CBAP may also activate integrins via an indirect mechanism. That is, CBAP may sequester 14-3-3 from binding to integrin β2, which would allow talin to freely bind to the β subunit of integrin and activate the integrin complex. On the other hand, the filamin protein family is implicated in signal integration to regulate cell mobility through interaction with various components, such as integrins, Vav2, and the chemokine receptors CXCR4, CXCR5 and CCR2, and so on [43], [45]–[49]. Using the yeast two-hybrid system and immunoprecipitation analysis, we found that filamin A is a candidate component associated with CBAP (unpublished data), lending some support to this notion.

In figure 4D and 4E, we showed that the basal levels of Rac1-GTP were lower in CBAP-knockdown C29 cells compared to control C21 cells, suggesting that CBAP may also control basal level of cell motility. Our unpublished data showed that filamin A preferentially binds to the inactive Rac1 mutant, Rac1-N17, instead of the active form of Rac1 (i.e., Rac1–V12). Furthermore, CBAP is required for optimal association of Rac1 with filamin A in unstimulated cells. Knockdown of CBAP reduced basal filamin A-bound Rac1level and chemokine stimulation further decreased it, suggesting once Rac1-GDP is converted to Rac1-GTP, it will dissociate from this filamin A/CBAP complex (unpublished data). CBAP also forms homodimers involving the N-terminus of each CBAP molecule (our unpublished data). Therefore, it is likely that CBAP dimers mediate Vav1-dependent GTP-for-GDP exchange on Rac1, thereby bringing filamin A–bound Rac1-GDP and the ZAP70-Vav1 complex together to facilitate the production of Rac1-GTP. More experiments are needed to support this hypothesis.

In conclusion, our study illustrates the importance of CBAP in regulating chemokine-dependent T-cell trafficking, including migration and adhesion. This role of CBAP may be due, at least in part, to its critical role in promoting chemokine-induced ZAP70/Vav1/Rac1 signaling and integrin activation.

## Supporting Information

Figure S1 **Targeted disruption of** ***CBAP*** **by homologous recombination.** (A) Schematic targeting strategy shows the genomic structures of the *CBAP* locus with two exons (open boxes) in the WT allele (*upper scheme*), targeting vector structure (*middle scheme*), and the chromosomal locus of the deleted allele after homologous recombination (*lower scheme*). Some relevant restriction enzyme sites (H3, HindIII; NI, NaeI), *loxP* sites (black filled triangles), the thymidine kinase (TK), neomycin resistance (Neo), and LacZ gene cassettes are indicated. The PCR primers used for genotyping analysis are as indicated (P1, P2 and P3). (B) Southern blot analysis of genomic DNA from the tail of mice with the indicated genotypes. The probe and predicted length of HindIII and NaeI restriction fragments are as indicated in panel A. (C) Detection of mRNA by RT-PCR in liver and purified CD90.2^+^ LN T cells from *CBAP* ^+/+^ and *CBAP* ^−/−^ mice. Gapdh mRNA was used as a positive control. (D) Immunoblotting (IB) of murine CBAP (mCBAP) in liver lysate of *CBAP* ^+/+^ and *CBAP* ^−/−^ mice.
(TIF)
