# Supplementary figures and images for: CBAP Functions as a Novel Component in Chemokine-Induced ZAP70-Mediated T-Cell Adhesion and Migration
Source: PLoS One. 2013 Apr 19;8(4):e61761. doi: 10.1371/journal.pone.0061761 (PMC3631140; doi:10.1371/journal.pone.0061761)

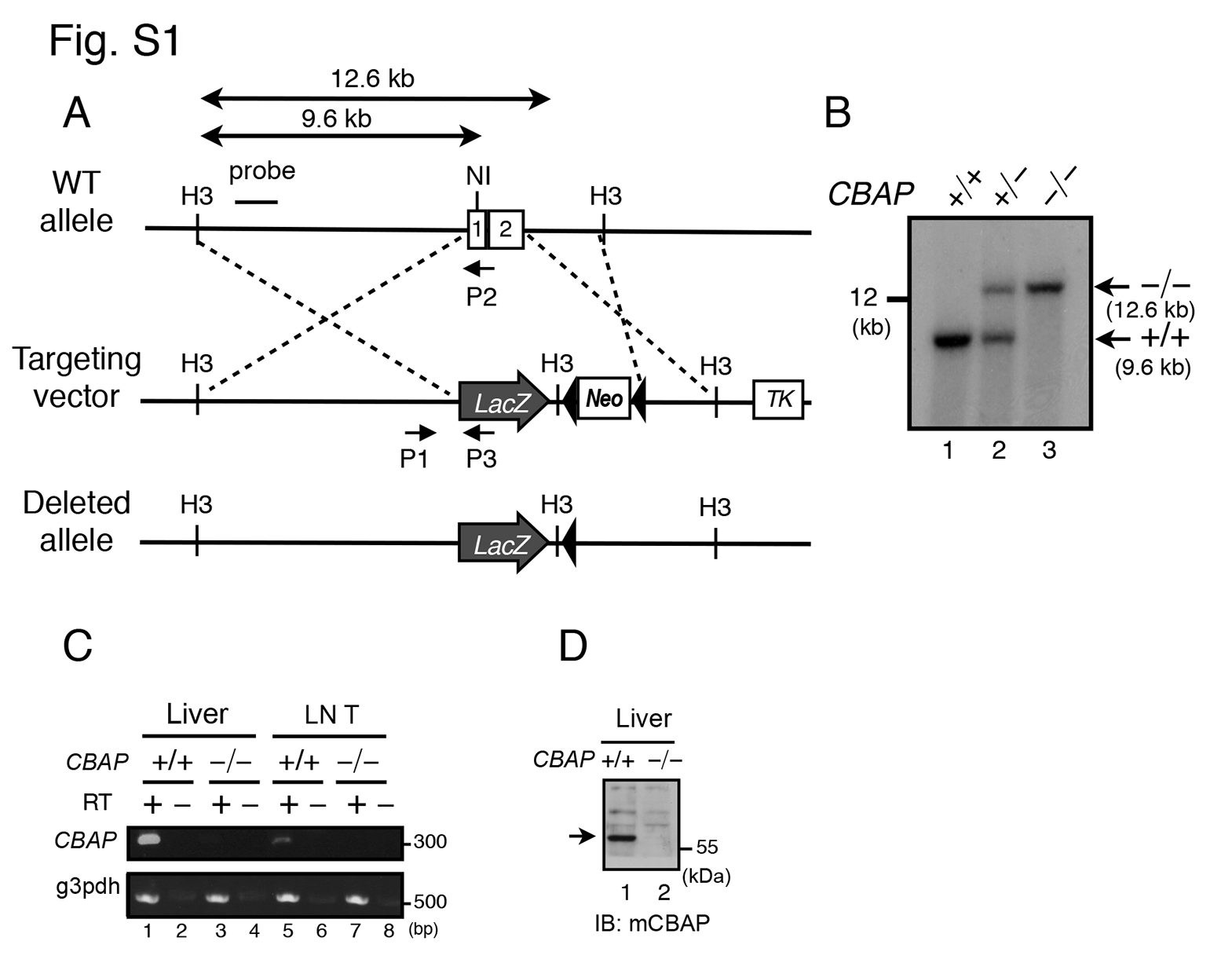

Supplement: Figure S1 — Targeted disruption of CBAP by homologous recombination. (A) Schematic targeting strategy shows the genomic structures of the CBAP locus with two exons (open boxes) in the WT allele (upper scheme), targeting vector structure (middle scheme), and the chromosomal locus of the deleted allele after homologous recombination (lower scheme). Some relevant restriction enzyme sites (H3, HindIII; NI, NaeI), loxP sites (black filled triangles), the thymidine kinase (TK), neomycin resistance (Neo), and LacZ gene cassettes are indicated. The PCR primers used for genotyping analysis are as indicated (P1, P2 and P3). (B) Southern blot analysis of genomic DNA from the tail of mice with the indicated genotypes. The probe and predicted length of HindIII and NaeI restriction fragments are as indicated in panel A. (C) Detection of mRNA by RT-PCR in liver and purified CD90.2+ LN T cells from CBAP +/+ and CBAP −/− mice. Gapdh mRNA was used as a positive control. (D) Immunoblotting (IB) of murine CBAP (mCBAP) in liver lysate of CBAP +/+ and CBAP −/− mice. (TIF) [file pone.0061761.s001.tif]
